# Curcumin: Overview of Extraction Methods, Health Benefits, and Encapsulation and Delivery Using Microemulsions and Nanoemulsions

**DOI:** 10.3390/ijms24108874

**Published:** 2023-05-17

**Authors:** Maria D. Ciuca, Radu C. Racovita

**Affiliations:** Department of Inorganic Chemistry, Physical Chemistry and Electrochemistry, Faculty of Chemical Engineering and Biotechnologies, University Politehnica of Bucharest, 1-7 Gh. Polizu St., District 1, 011061 Bucharest, Romania; maria_daniela.ciuca@upb.ro

**Keywords:** curcumin, bioactivity, extraction methods, microemulsion, nanoemulsion, encapsulation, delivery

## Abstract

Curcumin is the principal curcuminoid found in the rhizomes of turmeric. Due to its therapeutic action against cancer, depression, diabetes, some bacteria, and oxidative stress, it has been used widely in medicine since ancient times. Due to its low solubility, the human organism cannot completely absorb it. Advanced extraction technologies, followed by encapsulation in microemulsion and nanoemulsion systems, are currently being used to improve bioavailability. This review discusses the different methods available for curcumin extraction from plant material, methods for the identification of curcumin in the resulting extracts, its beneficial effects on human health, and the encapsulation techniques into small colloidal systems that have been used over the past decade to deliver this compound.

## 1. Introduction

**Figure 1:**
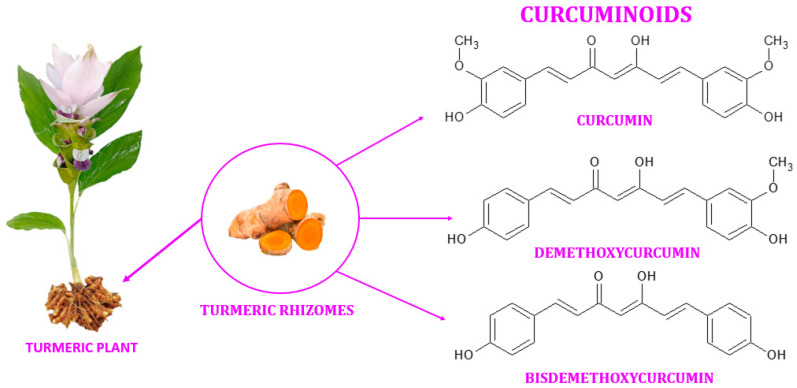
Curcuminoids found in the rhizomes of turmeric.

Turmeric (also known as Arab globe Curcuma, Haridra in Sanskrit, Chinese yellow ginger Jianghuang, Japanese Kyoo, or Ukon [1]) is a rhizomatous perennial plant [2] of the Zingiberaceae family [3] widely cultivated annually in tropical and subtropical regions of the world [4,5,6], the largest global producer of turmeric being India [1,4]. *Curcuma longa* is the most commonly recognized species within the genus *Curcuma*. However, other species exist within this genus, namely *Curcuma amada*, *Curcuma zedoaria*, *Curcuma aromatica*, and *Curcuma rakatakanta* [7]. At maturity, turmeric can reach a height of one meter. Its leaves are long, rectangular, and placed in two rows from which leaf sheaths and false stems, petioles, and leaf blades are formed. With a length between 2.5–7 cm and a diameter of approximately 2.5 cm, the rhizomes are roughly segmented and have a specific fragrance. Depending on the shape, two types of rhizomes are distinguished: the primary rhizomes that have a pear shape and are also known as bulbs, and the secondary rhizomes that have a cylindrical shape. The flowers are 10–15 cm long, have a matte yellow color, and are arranged in spike-like structures [8]. Since ancient times, this yellow spice has been used in the Asian medicine of Ayurveda (which emerged about 5000 years ago), Siddha, Atharveda (2000 years ago), Unani medicine, and Chinese medicine [9,10]. During the 14th century, the Western world was introduced to the golden spice by early European explorers of the Asian continent [11]. Further, it was also used in religious cult rituals [3,12]. It is also a natural spice used as a colorant in curry, mustard [13], salads, pasta, yoghurt, cheese, and baked goods [14]. It is referred to as Natural Yellow 3 as an ecological dye and has been given the E designation E100 when used as a food coloring ingredient [15]. Turmeric is made up of 69.4% carbohydrates, 13.1% moisture, 6.3% protein, 5.1% fat, and 3.5% minerals. The essential oil, with a proportion of 5.8%, contains, among other compounds, curcumin (C_21_H_20_O_6_) [12], a significant curcuminoid found in the rhizomes of turmeric that gives it its yellow color [16,17]. The other curcuminoids are demethoxycurcumin (C_20_H_18_O_5_) and bisdemethoxycurcumin (C_19_H_16_O_4_), which are present in lower concentrations (Figure 1) [18]. Depending on the geographical cultivation area, curcuminoids amount to around 30–150 mg/g of the turmeric rhizome [19]. Curcumin is biosynthesized from two molecules of feruloyl-CoA and one molecule of malonyl-CoA via two enzymatic conversions catalyzed by diketide-CoA synthase (DCS) and curcumin synthase. (CURS). DCS and CURS both belong to the family of type III polyketide synthases [20].

Curcumin derivatives have antioxidant, anti-diabetic, anti-cancer, anti-allergic, anticoagulant, anti-fungal, and anti-infertility properties [2,5,21,22,23]. However, the bioavailability of this bioactive compound is limited by its low solubility in water [24]. To increase its bioavailability, numerous researchers have tried multiple times and by different methods the encapsulation in micro and nano emulsions [25].

## 2. Chemistry of Curcumin

**Figure 2:**
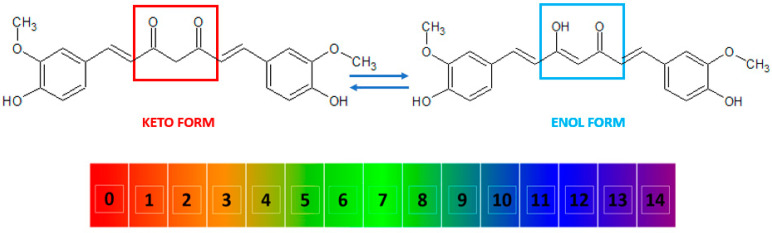
The keto and enol tautomers of curcumin and their reversible inter-conversion in aqueous medium as a function of pH. The numbers indicate the pH scale from 0 to 14. At low pH, the keto form (marked in red) is predominant, while at high pH, the enol form (marked in blue) predominates.

Curcumin, also known as diferuloylmethane [26], is a polyphenol from the group of diarylheptanoids with the IUPAC name (1*E*,6*E*)-1,7-bis(4-hydroxy-3-methoxyphenyl)-1,6-heptadiene-3,5-dione, a molecular weight of 368.39 g/mol, and the chemical formula C_21_H_20_O_6_ [16,27]. This compound has a symmetrical molecule that exhibits in its structure two phenyl rings substituted with a methoxy group in the ortho position and a hydroxy group in the para position. The two aromatic rings are interconnected by a seven-carbon chain consisting of an alpha-beta unsaturated diketone moiety [22], which makes it both a polyphenol and a polyketide at the same time [28,29,30,31]. The diketo group exhibits keto-enol tautomerism: in the solid state, it was shown to exist 100% in the enol form [32] and also predominate as an enol in alkaline aqueous solutions, while the keto form is predominant in acidic and neutral solutions, the enol representing only about 30% of all curcumin in the latter (Figure 2) [10,33,34].

Curcumin is characterized by the presence of three ionizable protons in aqueous solution, namely the enolic proton with a p*K*a value of approximately 8.5 and two phenolic protons with p*K*a values ranging from 10 to 10.5 [11]. The essential chemical reactions associated with the biological activity of curcumin are electron-donating reactions that lead to the oxidation of the compound, reversible and irreversible nucleophilic addition reactions, hydrolysis, and enzymatic reactions [12]. The degradation products of curcumin include ferulic acid, feruloyl methane, vanillin, and dicyclopentadiene [35]. Curcumin has a yellow color, a melting point of 183 °C [10], and a partition coefficient of logP ≈ 3, which is indicative that curcumin is a rather hydrophobic compound [4,12]. However, it is soluble in polar solvents such as dimethyl sulfoxide (DMSO), methanol, ethanol, and acetone [12].

## 3. Separation of Curcumin from Turmeric

The isolation of curcumin from the rhizomes of *C. longa* was initially carried out by Vogel and Pelletierin in 1815 [26,36]. Curcumin was first purified by Vogel Jr. in 1842 [26]. In 1910, Milobedeska et al. published a report on the structure of curcumin, identifying it as diferuloylmethane, following several decades of research. Lampe and Milobedeska successfully synthesized curcumin in 1913 [26,37]. In 1953, Srinvansen recorded the chromatographic separation and quantification of curcumin [26].

**Figure 3:**
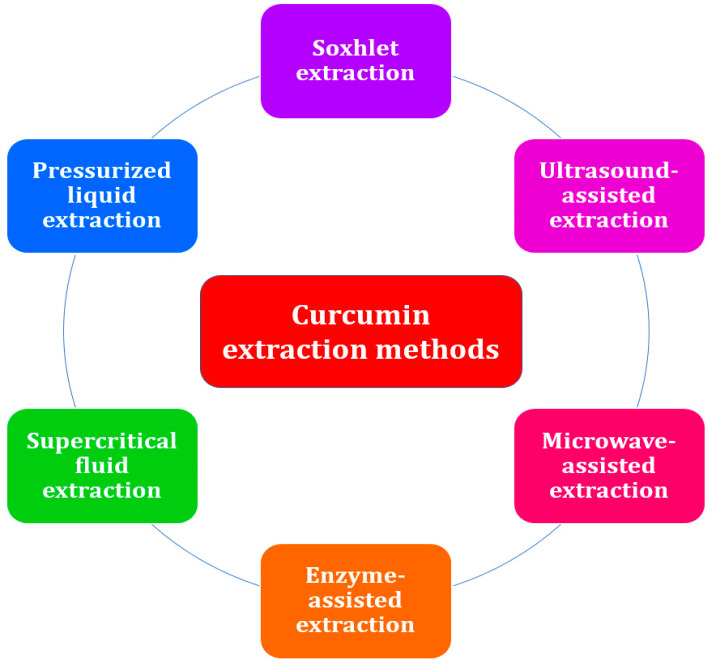
Conventional and advanced extraction methods for curcumin from plant material.

The process of curcumin extraction is a crucial step in the recovery of the bioactive compound from the plant matrix [38]. During the extraction process, specific solvents are used in accordance with predetermined procedures, leaving behind insoluble compounds [39]. Curcumin can be extracted using either conventional or advanced techniques. Since conventional extraction methods such as solvent extraction and Soxhlet extraction [7] require a considerable amount of time, organic solvents, cooling water [40], and energy [41], several researchers have applied novel extraction methods such as ultrasound-assisted extraction [42], microwave-assisted extraction [43], enzyme-assisted extraction [44], supercritical fluid extraction [45], and pressurized liquid extraction [46] (Figure 3). Regardless of the technique used, the preparation of the raw material prior to processing and the selection of the best parameters have a significant impact on the efficacy of the extraction technique and the quality of the extract. Typically, phytochemicals are more accessible and the matrix is more easily penetrated by the extraction solvent if the plant material is dried rather than fresh and finely ground as opposed to simply cut into pieces. The solvent-to-plant solid material ratio can also be decreased when the latter is finely pulverized, thus reducing costs [38]. Recirculating the solvent as in a Soxhlet extractor can also lead to the processing of a greater amount of material with less solvent cost and increased extraction yields, as can intense agitation, ultrasounds, microwaves, specific enzymes, or high pressures, as described below.

### 3.1. Solvent Extraction

Solvent extraction is the continuous or batch mass transfer of the target compound into the polar or nonpolar extraction solvent [47]. The first stage of this process involves the penetration of the crushed plant solid sample by the solvent, typically ethanol, methanol, isopropanol, or acetone; in the second stage, the solute of interest is dissolved in the solvent, and in the next stage, it is rereleased outside the solid matrix. The last step consists of collecting the solute [48]. The phenomena of solid matrix penetration and solute migration occur simultaneously until equilibrium is reached [47]. The solvent is removed by evaporation at the end of the process to obtain a high-quality concentrated product [49].

### 3.2. Soxhlet Extraction

In 1879, German chemist Franz Ritter von Soxhlet invented the Soxhlet extractor for lipid extraction. Nowadays, this device is also used to extract bioactive compounds from natural sources [50]. The experimental setup for soxhletation consists of a heating nest, a distillation flask, a Soxhlet extractor, and a condenser. The dried sample is introduced into the thimble and placed in the Soxhlet extractor. By heating the distillation flask, the solvent changes into a vapor state. Then it is condensed and introduced into liquid form over the thimble in the extractor. The solvent penetrates the cellular wall of the solid sample, and the compound of interest is extracted. When the solvent volume reaches the extractor’s overflow level, the liquid is aspirated by a syphon and returned to the distillation flask. The extraction cycle is repeated until the bioactive compound of interest reaches the desired concentration [51,52].

Soxhlet extraction is nowadays used as a reference extraction method, sometimes leading to extraction yields of curcumin close to 100%; however, it is a lengthy process and requires high energy costs [53].

### 3.3. Ultrasound-Assisted Extraction

Ultrasound-assisted extraction (UAE) is an environmentally friendly extraction technique. This method is based on the phenomenon of cavitation [54]. Ultrasound is defined as sound waves with frequencies ranging from 20 kHz to 100 MHz that cannot be detected by human ears [55]. Ultrasonication is extensively used in industry and is referred to as a “green unique technology” due to its contribution to environmental sustainability [56]. Depending on the amplitude and frequency of the sound waves [57], the mechanism can be thermal or athermal. In the case of the thermal mechanism, the energy absorbed at the impact with the sound wave is converted into heat [55]; in the case of the athermal mechanism, the passage of the sound waves through the liquids leads to the formation of gas bubbles due to repeated cycles of compression and expansion [54]. Due to the Brownian motion, the bubbles accumulate energy, and when they burst, they transmit the energy to the cell wall of the plant and the extraction medium, thus causing the breaking of the intermolecular bonds between the compounds of interest and the solid matrix [53]. This method is advantageous due to its short extraction time and low solvent and energy consumption [58]. Wimonrut et al. achieved an extraction yield of 160.3 mg/g by utilizing ethanol as the solvent, employing a solid-liquid ratio of 1:10, operating at a frequency of 42 kHz, applying 240 W input power, and conducting the extraction process for a duration of 40 min [59]. Shisath et al. studied the variation of different extraction parameters to find the optimum conditions for the extraction of curcumin using ultrasound-assisted extraction. They found that the optimum extraction parameters were 40 °C as temperature, 1:30 as solid to solvent ratio, 0.09 as the mean particle size, 240 W as ultrasonic power, 22 kHz as frequency, and ethanol as the most suitable solvent. Using the optimum conditions, the extraction yield was 73.18% in 2 h, as compared to Soxhlet, where the extraction yield was 100% but the parameters were 78 °C as temperature and 14 h as extraction time [53].

### 3.4. Microwave-Assisted Extraction

Microwave-assisted extraction (MAE) is based on the energy transfer from microwave radiation to the cell wall [60]. Electromagnetic waves with frequencies between 300 MHz and 300 GHz are known as microwaves [50]. When microwave radiation strikes a dielectric material [60], ion conduction and dipole rotation mechanisms turn the energy into heat [50,57]. The plant moisture is heated, which puts pressure on the cell wall until it is broken and the bioactive compounds are released [50,61]. The three sequential steps of the microwave-assisted extraction mechanism are: (1) separating the solutes from the sample matrix active sites under increased pressure and temperature; (2) diffusion of the solvent throughout the sample matrix; and (3) releasing the solutes from the sample matrix into the solvent [50].

Recently, Singh et al. [57] obtained curcuminoids using novel extraction methods such as MAE, UAE, and Soxhlet extraction as a traditional method. The results show that the novel extractions have higher total curcuminoid contents than the traditional ones. Curcuminoid content in MAE, UAE, and Soxhlet, for instance, was 326.79, 241.17, and 215.83 mg/g, respectively. This research also reported that MAE is more environmentally friendly because it requires less heating, has lower extraction costs, and has a higher extraction yield [57]. In the study of Fernandez-Marin et al. [62], curcumin was extracted by MAE: the conditions were 29.99 min, 160 W, a solid/solvent ratio of 1:20 g/mL, and the yield of extracted oil obtained was 10.32 ± 0.17%. The yield value was higher than that obtained by a conventional Soxhlet method (8.44 ± 0.17%) [62].

### 3.5. Enzyme-Assisted Extraction

This extraction method is based on hydrolytic enzymes that degrade the polysaccharide polymers of the cell wall. Since target metabolites are also kept in the wall matrix by hydrogen or hydrophobic bonds, they are released from both the internal cell environment and the cell wall [63]. Commonly used enzymes for this process are lipase, amylase, pectinase, amyloglucosidase, lactase, and protease [64]. The factors influencing the extraction yield are pH, enzyme concentration, enzyme type, incubation time, and temperature [63,65]. Although it is an environmentally friendly process, the long extraction time presents a significant disadvantage to this method [64].

### 3.6. Supercritical Fluid Extraction

Supercritical fluid extraction (SFE) involves raising the temperature and pressure of the extractant above its critical points [66]. CO_2_ is frequently used as a solvent because it has a low critical temperature and is odorless, colorless, and non-toxic [67]. The extraction process is divided into four stages: the first stage involves the diffusion of the supercritical fluid into the porous matrix of the sample; the second stage consists of the equilibration of the sample and the solvent; the third stage involves the diffusion of the solute out of the matrix; and the final stage consists of the recovery of the analytes via decompression [68]. Due to its low temperature, this procedure is appropriate for the extraction of thermally stable and easily oxidized compounds [66]. Recently, Widmann et al. [69] obtained curcuminoids by SFE using carbon dioxide as solvent at a constant temperature (75 °C) and pressure (425 bar). The flow rate was 0.5 kg/h for 1 h. The maximal recoveries yielded were 0.68–0.73% [69].

### 3.7. Pressurized Liquid Extraction

Pressurized liquid extraction (PLE) relies on elevated temperature and pressure conditions that promote the desorption and solubilization of analytes into different solvents [70]. Water is one of the solvents that can be used in this process, and it can be kept in the liquid phase at temperatures ranging from 100 to 374 °C with pressures sufficiently high to avoid phase transitions [71]. As a result, dielectric properties change, the diffusion rate increases, viscosity decreases, and surface tension decreases, and thus it functions similarly to an organic solvent [66].

Hye-Lin Kwon and Myong-Soo Chung [72] studied the extraction of demethoxycurcumin, bisdemethoxycurcumin, and curcumin under a variety of conditions, including temperature (110–150 °C), time (1–10 min), pressure (5–100 bar), solid to solvent ratio, and solvent mixing ratios. Before the extraction, turmeric was dried with hot air and then cut into pieces. The sample was kept at 4 °C. Variable extraction was used after 1 g of the sample was added to a solvent that contained 50% water and 50% ethanol. They discovered that the ideal extraction parameters were 135 °C, 5 min, and a mixture ratio of 1:20 (*w*/*v*) for the solid and solvent. Under optimal conditions, the concentration of curcuminoids was 15.8%.

In another study, the curcumin extraction time by the PLE process was compared with the Soxhlet extraction process. The rhizomes were first deflavored by supercritical extraction with CO_2_. Following this step, ethanol was used as solvent for the PLE process, which was carried out with a fixed extraction time of 20 min and independent variables of pressure (100–300 bar) and temperature (60–77 °C). According to the results, the optimal extraction temperature and pressure were 60 °C and 100 bar, respectively. To achieve comparable extraction yields, PLE required an extraction time three to six times shorter than Soxhlet extraction [73].

Table 1 contains a summary of all the different extraction methods for curcuminoids discussed, including the essential experimental details and extraction yields attained.

## 4. Methods for Identification and Characterization of Curcumin in Plant Extracts

The resulting curcumin extracts have been analyzed for the presence and content of curcuminoids by various analytical methods, which primarily consist of spectroscopic methods and chromatographic methods (often involving spectrometric analyzers), but also, albeit less frequently, other modern methods, such as electrochemical, nanosensor, or calorimetric methods.

### 4.1. Spectroscopic Methods

Among the spectroscopic methods employed to characterize curcumin and related compounds are ultraviolet-visible (UV-VIS) spectrophotometry, Fourier transform infrared (FTIR) spectroscopy, fluorescence spectroscopy, and nuclear magnetic resonance (NMR) spectroscopy.

Direct measurement of the UV-VIS absorbance spectra of plant-based extracts, typically performed in methanol or ethanol [82], has found limited applicability for the unequivocal identification of curcumin due to interference from other absorbing species coextracted from turmeric rhizomes. Nonetheless, it is a suitable method when only a rough estimation of curcuminoids suffices. In such cases, absorbance is usually measured at or around 425 nm in an alcoholic solution [83].

Similarly, FTIR spectroscopy has the same drawbacks as UV-VIS and has very rarely been used for characterizing the composition of turmeric extracts. The direct application of this technique on plant extracts demands its combination with chemometric methods such as principal component analysis (PCA) [84] or partial least-squares regression [85], which usually implies a very large number of samples [86]. The application of such methods has made it possible to discriminate compositions of extracts of turmeric (*C. longa*) and java turmeric (*C. xanthorrhiza*) [84,87]. When preceded by additional purification steps, plant-derived, purified curcumin can be identified by FTIR fingerprinting. Thus, from the ethanolic rhizome extract, evaporated at 50 °C in a vacuum rotary evaporator and then fractioned twice using *n*-hexane, curcumin was characterized by FTIR and fingerprinted against an authentic standard, retrieving all its characteristic bands at 3229 cm^−1^ (broad, OH stretching vibration), 2969 and 2922 cm^−1^ (due to CH_3_ and CH_2_ stretching, respectively), 1628 cm^−1^ (C=O stretching), 1585 cm^−1^ (C=C stretching), 1427 cm^−1^ (CH_2_ bending), 1338 cm^−1^ (CH_3_ bending), and 1030 cm^−1^ (C-O stretching) [88]. Similar FTIR absorption bands were also reported by Perera et al. [85].

Fluorescence spectroscopy has also been quite infrequently used for the detection of curcumin in samples of plant origin. Curcuminoids were shown to exhibit fluorescence emission spectra typically ranging from about 548 to 575 nm, with curcumin in particular having an emission maximum at 571 nm when excited at the 467 nm excitation wavelength [89]. The emission maximum was influenced by humidity, as it shifted to 550 nm when dry turmeric powder was analyzed as opposed to wet fresh rhizomes [89].

^1^H-NMR spectroscopy is also limited by the potential overlap of numerous proton signals of organic origin that may come from other plant extract materials. Vitasari et al. [90] managed to isolate curcumin from the rhizome extract of *Curcuma soloensis* Val. by Soxhlet extraction with acetone, followed by evaporation to dryness, fractionation, and purification by vacuum liquid chromatography using an n-hexane:ethyl acetate eluent. Curcumin was identified by its bright orange color and by its characteristic ^1^H-NMR signals: two highly deshielded alkene protons at 7.59 ppm, six aromatic protons between 6.93–7.15 ppm, two other alkene protons at 6.48 ppm, one enol alkene proton at 5.81 ppm, and six methoxy protons centered around 3.95 ppm [90].

### 4.2. Chromatographic Methods

Among the chromatographic techniques used for natural curcuminoid analysis are liquid chromatography (LC), gas chromatography (GC), and thin layer chromatography (TLC). By far the most commonly employed analytical technique for the identification and quantification of curcumin is liquid chromatography, usually coupled with UV-VIS absorption analyzers or various mass spectrometers (MS).

Vidal-Casanella et al. [91] extracted into methanol the natural products from turmeric and commercial curry samples using UAE for 40 min and then separated them by high-performance liquid chromatography with a diode array detector (HPLC-DAD). A reversed-phase protocol was employed using a X Terra MS C18 column and a gradient elution with a mobile phase containing between 40 and 90% acetonitrile in water and 0.1% formic acid. Curcumin was monitored using its fingerprint UV-VIS absorption at 420 nm [91].

Similarly, Wulandari et al. [88] analyzed the curcuminoids from the ethanolic extract of *C. longa* obtained after 24-h maceration in 90% ethanol by HPLC-PDA (photodiode array) on a Waters X-bridge C18 reversed-phase column using an acetonitrile-aqueous acetic acid (2%) mobile phase delivered isocratically at 30 °C. In this case, the absorption of curcuminoids was monitored at 425 nm [88].

Nunez et al. [92] performed the profiling of curcuminoids extracted into DMSO by UAE from 21 turmeric (*C. longa* and *C. zedoaria*) varieties and from 9 curry samples by targeted LC-HRMS (high resolution mass spectrometry). This was performed on a reversed-phase porous shell Ascentis Express C18 column with gradient elution using a water-acetonitrile mobile phase with 0.1% formic acid from a low 10% to a high 95% acetonitrile content. Curcuminoids were identified based on their high-resolution mass spectra obtained with a Q-Orbitrap mass analyzer operated in negative ionization mode.

HPLC coupled with tandem mass spectrometry (HPLC-MS/MS) was also successfully employed to separate and discriminate between four different curcuminoids (curcumin, demethoxycurcumin, bisdemethoxycurcumin, and tetrahydrocurcumin) on a Zorbax Extend C-18 3.5 µm Agilent Column, maintained at 35 °C, and operated under gradient elution with an aqueous 0.1% formic acid-methanol eluent containing from 50 to 95% methanol. The unequivocal assignment of structures was done using an AP Sciex API4000 mass spectrometer based on the exact masses of molecular and product ions of each compound: 369.4/177.1 Da (for curcumin), 339.1/119.0 Da (for demethoxycurcumin), 309.4/199.3 Da (for bisdemethoxycurcumin), and 373.2/137.2 Da (for tetrahydrocurcumin) [93].

Gas chromatography (GC) has very rarely been used as an identification and characterization method for curcumin due to the reduced volatility of this compound. When a flame ionization detector (FID) is coupled to the GC, identification of curcumin is only possible when an authentic standard is also available for comparison of retention times or retention indices of the natural product and the standard, respectively [94]. In most cases, however, identification is facilitated by coupling the GC to a mass spectrometer (GC-MS), which provides a fingerprint mass spectrum in addition to retention time or index. In this manner, Nsofor et al. [95] identified curcumin in the concentrated methanolic extract of *Tetrapleura tetraptera* alongside other phytochemicals using an Agilent 6890N GC equipped with a DB-5ms capillary column and helium as carrier gas in conjunction with an Agilent 5975B mass spectrometer detector (MSD). Using similar conditions, Noori et al. [94] identified curcumin among several secondary metabolites in the Soxhlet ethanolic extract of *Rheum ribes* based on its characteristic m/z values of 368.3 Da (corresponding to the parent ion) and 254 Da (corresponding to a product ion).

Kushwaha et al. [96] performed high-performance thin-layer chromatographic (HPTLC) analysis of the methanolic fraction from the extract of *Curcuma longa* L. rhizomes on plates made of silica gel 60 stationary phase and using a chloroform-methanol 97:3 (*v*/*v*) mobile phase. The developed plates were then visualized with a deuterium lamp at 420 nm to reveal the presence of curcumin [96]. In addition, using HPTLC but with a different mobile phase consisting of toluene-ethyl acetate-formic acid 9:6:0.4, Taha et al. separated curcumin from the ethanolic fraction of the turmeric rhizome extract but visualized it at 410 nm, attributing the slightly different absorption maximum to pH variations [97].

### 4.3. Other Methods

More recently, other analytical methods have started to find application in the detection of curcumin and curcuminoids from plant extracts.

Among electrochemical techniques, capillary electrophoresis (CE) with PDA detection was successfully applied to separate and identify curcuminoids and their degradation products from turmeric rhizomes extracted with ethanol by a UAE protocol [98]. Later, Wu et al. upgraded the method to a micellar electrokinetic chromatographic version coupled with laser-induced native fluorescence detection and thus improved the detection of all three curcuminoids in turmeric, medicinal turmeric liniment, and curry seasoning [99]. Other approaches involved the development of graphite electrodes embedded into molecularly imprinted polymers, which served for the determination of curcumin in various buffers by either cyclic voltammetry or differential pulse voltammetry [100].

Nanosensors for curcumin are compact devices usually based on either spectrofluorimetric or electrochemical detection [83]. Fluorescent nanomaterials such as lanthanide nanoparticles, Mn-doped ZnS quantum dots, metal nanoclusters, and carbon quantum dots have been used as chemical probes for curcumin [101]. Carbon dots (CD) are most frequently used as their fluorescence can be easily quenched by curcumin, proportionally to its concentration. To improve their fluorescence quantum yield, CDs were doubly doped with nitrogen and phosphorus when prepared from glucose as a carbon source [102] or with nitrogen and boron when prepared from citric acid monohydrate [103]. Both approaches led to substantial improvements in curcumin detectability.

Thermochemical methods were also very recently introduced as an option for curcumin identification. Opustilova et al. [104] managed to apply modulated differential scanning calorimetry (MDSC) for the identification of curcumin in its solid state. The characteristic melting point of curcumin crystals was indicated by an endothermic peak with an onset temperature of 168.92 ± 0.11 °C, a peak temperature of 174.83 ± 0.07 °C, and a specific enthalpy of fusion equal to 80.83 ± 0.15 J/g [104].

## 5. Health Benefits of Curcumin

After ingestion, curcumin is metabolized mainly in the liver, large intestine, and intestinal microbiota [105,106,107]. The metabolism process is carried out by enzymes and occurs in two phases: the first phase involves the reduction of the four double bonds of the heptadiene-3,5-dione [108,109] structure in heterocytes and hepatocytes by a reductase to dihydrocurcumin, tetrahydrocurcumin, hexahydrocurcumin, and octahydrocurcumin [105,106,107]. In the second phase, unmetabolized curcumin and the resulting metabolites from the first phase are transformed into glucuronidic and sulphate-O-conjugated metabolites because glucoronidases and sulphatransferases can conjugate the glucuronic acid and sulphate molecules to hydroxyl groups [107,108]. Numerous clinical studies have suggested curcumin has antioxidant, antidiabetic, antibacterial, antidepressant, and anticancer properties and beneficial effects on diabetes mellitus and other disorders [110].

### 5.1. Antioxidant Properties

Reactive oxygen species (ROS) are oxygen-derived molecules with short lifetimes and high reactivity due to the presence of unpaired valence electrons [111]. Both endogenous (such as mitochondria, peroxisomes, endoplasmic reticulum, etc.) and exogenous (such as pollution, alcohol, tobacco smoke, heavy metals, medications, etc.) sources contribute to the production of free radicals [112]. The primary source of ROS production is the mitochondria, where adenosine triphosphate (ATP) production by oxidative phosphorylation takes place with the reduction of molecular oxygen to H_2_O in the electron transport chain [113]. Although they are by-products, they play an essential role in cell signaling and homeostasis by regulating cell proliferation, differentiation, and survival [114].

The concept of oxidative stress was defined as “an imbalance between oxidants and antioxidants in favour of the oxidants, leading to disruption of redox signalling and control and/or molecular damage” in “Oxidative stress: a concept in redox biology and medicine” [115]. The best-known ROS are peroxides, superoxides, hydroxyl radicals, ozone, and nascent oxygen molecules. In high concentrations, these species have the ability to oxidize nucleic acids, lipids, and proteins. Antioxidants scavenge ROS by donating their electrons to the undervalent ROS and neutralizing it.

Curcumin can reduce mitochondrial oxidative stress by increasing the effects of superoxide dismutase, glutathione, and catalase. The three redox sites of curcumin can undergo oxidation and hydrogen abstraction, resulting in the formation of phenoxy radicals and stabilization against the enol structure [116].

Curcumin at 645 mg/24 h for 67 days boosted total antioxidant capacity and lowered malondialdehyde levels, according to a meta-analysis of 308 individuals, 60% of whom were women [117]. In an in vivo investigation, the cardioprotective and hepatoprotective properties of curcumin against hepatotoxicity and cardiotoxicity generated by doxorubicin (DOX), a medication often used in tumour disorders, were studied. To elicit unfavourable effects, mice were given a single dose of DOX (20 mg/kg) intraperitoneally. Curcumin (100 mg/kg oral) was administered to them 10 days before and 5 days after DOX treatment. Due to its anti-inflammatory and antioxidant properties, curcumin reduces lipid peroxidation, inhibits immunoexpression of nuclear factor-kB (NF-kB), inhibits tumour necrosis factor-alpha (TNF-α) and inducible nitric oxide synthase (iNOS), and lowers circular inflammatory interferon-gamma (IFN-γ) levels. As a result, it was revealed that curcumin may be administered as an adjuvant since it can serve as a protective antioxidant-based method against DOX-associated cardiotoxicity and hepatotoxicity [118].

### 5.2. Antibacterial Properties

Due to their both beneficial and harmful impacts, the existence of microbes is well known in the human population. When the symbiotic relationship between microbes exceeds a certain threshold, they can produce pathogenic illnesses and diseases that can be fatal to the human body. Antimicrobial agents, particularly antibiotics, are used to combat bacterial infections [119]. Bacteria have evolved and developed resistance to antibiotics throughout time as a result of their overuse or inadequate usage [120]. Antibacterial antibiotic resistance is one of the leading causes of treatment failure. Traditional medicines have been shown to have a considerable impact on pathogen therapy by regulating a variety of physiological systems [121].

The antibacterial activities of curcumin were initially documented by Schraufstatter and colleagues in 1949 [122]. Despite its poor solubility in water, limited bioavailability, and pharmacokinetic profile, modern investigations have shown high antibacterial activity for curcumin [123]. The antibacterial mechanism of action of curcumin involves damage to the cell membrane, interference with cellular processes by targeting DNA and proteins, and inhibition of bacterial quorum sensing [124].

Maleki Dizaj et al. [125] studied the antibacterial effects of curcumin nanocrystals against *Posphyromones gingivalis* (*P. gingivalis*) isolated from the gingival crevicular fluid of Iranian patients with implant failure. The disc diffusion method was utilized to test bacterial sensitivity to curcumin nanoparticles, and the broth microdilution method was employed to estimate the nanoparticles’ minimal inhibitory concentrations (MICs) against *P. gingivalis*. The bacteria were sensitive at 50, 25, 12.5, and 6 µg/mL concentrations. At 50 µg/mL, curcumin nanocrystals demonstrated the most significant inhibitory zone. According to the MIC test, curcumin nanoparticles inhibited *P. gingivalis* growth at 6.25 µg/mL. Curcumin nanoparticles had bactericidal activity against *P. gingivalis* at 12.5 µg/mL in the MBC test (minimum bactericidal concentration) [125]. In another study, Snetkov et al. evaluated the antibacterial properties of polymer nanofibers based on hyaluronic acid and curcumin against multidrug resistant ESKAPE pathogens (*Enterococcus faecium*, *Staphylococcus aureus*, *Klebeciella pneumoniae*, *Acinetobacter baumannii*, *Pseudomona aeruginosa*, and *Enterobacter species*). The HA-curcumin stable complex showed high antibacterial activity against Gram-positive and Gram-negative bacteria. The minimum inhibitory concentrations for Gram-positive bacteria reached 90 µg/mL, while those for Gram-negative bacteria ranged from 90–960 µg/mL [126].

### 5.3. Antidepressant Properties

Depression is a psychiatric condition that affects about 300 million people annually, resulting in over 800,000 deaths. This illness costs Europe EUR 92 billion annually, with a substantial proportion of the financial burden imposed by the productivity loss of affected persons [127]. Fatigue, anhedonia, sleep difficulties, and self-destructive behaviour are the most common symptoms [128]. During the SARS-CoV-2 epidemic, prevention efforts led to social isolation, economic instability, and worries about losing loved ones, resulting in both physical and mental health deterioration [129,130,131,132]. Some prevalent antidepressant medicines have been linked to a number of negative side effects, prompting their early withdrawal [133]. These effects include constipation, dry mouth, sleep difficulties, cardiotoxicity, neurotoxicity, orthostatic hypotension, and sexual dysfunction [134].

Disruptions in the intestinal microbiota and disruptions of the gut-brain axis are significant factors in the development of depression. Depression episodes may be induced by a reduction in neurotrophic factors derived from the brain and a change in the efficiency of neurotransmitters due to changes in the permeability of the intestinal barrier caused by changes in the variety of the intestinal microbiota [128]. Since the intestine and liver are the primary sites of curcumin metabolism, it may be acting on the gut microbiota to reduce intestinal inflammation and act as a neuroprotective agent because neuroinflammation is likely a factor in many psychiatric disorders [135].

Inhibition of the monoamine oxidase (MAO) enzyme, modulation of the levels of different neurotransmitters (norepinephrine, dopamine, and serotonin), promotion of hippocampal neurogenesis, and its use as an anti-inflammatory agent are just a few of the details about the phenomenon of curcumin that support its use in treating major depression [136]. A 12-week research study was carried out by Kanchanatawan et al. to assess the benefits of adjunctive curcumin for treating major depressive disorder (MDD). The 65 MDD patients in the double-blind, placebo-controlled experiment were randomized to receive either adjunctive curcumin or a placebo. They found that adding curcumin to the medication considerably improved the Montgomery–Asberg depression rating scale (MADRS) score; this impact was significant 12 weeks after commencing treatment, and the benefit was maintained four weeks after curcumin withdrawal. With substantial differences between curcumin and placebo at weeks 12 and 16, curcumin was considerably more effective than placebo at alleviating depressive symptoms in severe depression. A greater effectiveness of curcumin was reported in males compared to females [137]. In mice, Qi et al. studied the antidepressant effects of curcumin administered nasally. They created a thermosensitive hydrogel that contained curcumin. After administering it, they noticed a rise in the levels of norepinephrine, dopamine, and 5-hydroxytryptamine, as well as its metabolites, in the striatum and hippocampus, proving that curcumin has antidepressant effects [138].

### 5.4. Diabetes Mellitus

Diabetes mellitus is a metabolic condition defined by persistent hyperglycemia in which insulin hormone activity on cell receptors is absent or inefficient, resulting in a substantial rise in blood glucose [139]. The types of diabetes mellitus according to the classification by the American Diabetes Association are: (I) type 1 diabetes, with autoimmune β-cell destruction and absolute insulin shortage; (II) type 2 diabetes (90–95% of diabetes cases), which is characterized by insulin resistance, followed by insulin hypersecretion in β cells of Langerhans, preventing the body from using its own insulin [140,141]; (III) gestational diabetes mellitus, which affects 7% of pregnancies in the second or third trimester; (IV) other kinds of diabetes, such as monogenic diabetes syndromes, exocrine pancreas illnesses, and drug- or chemical-induced diabetes, which account for 5% of diabetes patients [141]. Obesity and overweight are two main risk factors for diabetes caused by lifestyle choices [142]. Hyperglycemia, glucotoxicity, and oxidative stress are all linked to diabetes. Together, these things make advanced glycation endproducts (AGEs) and lipid peroxidation products, which increase ROS production in the cell [143]. Many organs, such as the eyes, nerves, heart, kidneys, and blood vessels, are susceptible to long-term damage, dysfunction, and failure due to diabetes [144].

According to studies conducted on diabetic patients, curcuminoids enhance insulin resistance, decrease levels of leptin, resistin, interleukin (IL)-6 IL-1β, and tumor necrosis factor-α, increase the release of adiponectin, and decrease glucose and insulin levels. Consequently, these compounds may influence glucose homeostasis and the consequences of diabetes [145]. A study conducted on male Long-Evans Tokushima Fatty Otsuka rats and Long-Evans Tokushima Otsuka rats (LETO controls) revealed that combining exercise with a curcumin-based diet (5 g/kg) improves glucose homeostasis and lipid profiles, promotes weight loss, and reduces levels of inflammatory response indicators IL 6, TNF, IL10, and ER stress marker levels. Using a Morris water maze test, they also assessed cognitive performance and discovered that curcumin boosts memory retention and escape latency [146]. In another study, the effect of curcumin on alpha-amylase was investigated in rats administered with 10, 20, 40, and 80 mg/kg of curcumin for 30 days. Blood glucose levels were monitored once every three days, while insulin levels were assessed on the first, middle, and final days of the trial. According to the data, curcumin inhibits alpha-amylase with an IC50 equal to 51.32 µM and an inhibition constant K_i_ of 20.17 µM, as well as decreasing glucose and insulin levels [147]. AMPK (5′-adenosine monophosphate-activated protein kinase) may alter metabolic phenotypes from fat synthesis to fat oxidation, reducing hepatic glucose production and increasing muscle glucose absorption. Lu et al. revealed that curcumin controls hepatic oxidative stress (thiobarbituric acid reactive substances, superoxide dismutases, glutathione, and catalase) and activates AMPK in gestational diabetic mice [148].

### 5.5. Anticancer Properties

Cancer is the leading cause of mortality globally, with approximately 10 million deaths recorded in 2020 and an estimated 28.4 million new cases expected by 2040 [149]. The lack of physical activity, obesity, tobacco use, alcohol use [150,151], exposure to ultraviolet radiation [152], and exposure to an environment polluted with nitrogen dioxide [153], sulfur dioxide, carbon monoxide [154,155], suspended particles [156], and persistent organic pollutants (POP) such as polycyclic aromatic hydrocarbons [157,158], all contribute to the development of various types of cancer, the most prevalent of which are breast cancer [159], colorectal cancer [36,160], lung cancer [154], prostate cancer, and gastric cancer [161].

In order to eradicate the disease or slow down its rate of spreading, several therapies can be used, including surgery, radiotherapy, chemotherapy, and immunotherapy [15,149,162]. Although there are a variety of treatments, they have side effects and limited effectiveness [149]. There are numerous studies highlighting the anticancer effects of curcumin, which come from the fact that curcumin regulates cell signalling pathways such as STAT3, NF-kB, activated Egr-1, AP-1, P53 [163], Wnt/β-catenin, PI3K/Akt, JAK/STAT, and MAPK [164].

Li et al. [165] explored the effect of curcumin against colorectal cancer in an in vitro investigation. The results revealed that curcumin was not hazardous to epithelial cells of the colonic mucosa, that it reduced colon cancer cell growth, and that it promoted apoptosis via a P53-mediated mechanism, upregulation of pro-apoptotic proteins such as Bax, and cell cycle arrest during S phase by inhibiting cell-cycle-related protein production, phosphorylation of Rb signalling pathway proteins, and E2F family transcription factors [165]. The anticancer effects of curcumin on multicellular breast cancer spheroids were studied by Kamalabadi et al. Breast cancer stem cells (BCSCs) were evaluated in vitro using spheroids. Three-dimensional (3D) culture methods were chosen because they better maintained the biological properties of the original malignancies than typical two-dimensional (2D) monolayer cultures. Curcumin induces death in breast cancer cells (MCF-3) in 3D culture, according to this study. This bioactive substance drastically reduced MCF-3 cell survival in 2D and 3D systems in a dose- and time-dependent manner; nevertheless, curcumin’s impact has been limited due to its low bioavailability [166]. In a recent study, Bolat et al. developed curcumin-loaded emulsions that increase the bioactive compound’s solubility by up to 10,000 times and extend the therapeutic effect against hepatocellular carcinoma. These loaded emulsions were evaluated in vitro on the androgen-dependent prostate cancer cell line LNCaP, and a substantial drop in androgen receptor gene expression levels was observed after treatment [167].

### 5.6. Side Effects and Potential Toxicity

The United States Food and Drug Administration (FDA) has acknowledged the general safety of curcumin, with its LD_50_ value determined in rats, i.e., the lethal dosage expected to cause the death of 50% of target species subjects [168], being well above 2 g/kg body weight [169]. The Joint Food and Agriculture Organization/World Health Organization (FAO/WHO) expert committee on food additives has established an acceptable daily intake (ADI) range of 0.1–3 mg/kg body weight for this substance [170,171].

Chanaken et al. conducted an in vivo study wherein they observed that the administration of curcumin at high doses and for prolonged periods (100 mg/kg/90 days) resulted in an imbalance in rats. This imbalance was characterized by an overproduction of reactive oxygen species (ROS), an increased production of the pro-oxidant cytokine IL6, and a decrease in antioxidant enzymes. These changes ultimately led to oxidative stress-mediated liver injury and inflammatory disorders [172].

In another study, fifteen people diagnosed with advanced colorectal cancer ingested curcumin-compatible capsules, with dosages ranging from 0.45 to 3.6 g per day, for a maximum duration of four months. Gastrointestinal adverse effects were reported by the patients. Two patients who were administered 0.45 g and 3.6 g of curcumin per day, respectively, experienced diarrhea (grades 1–2). A single patient who ingested a daily dose of 0.9 g of curcumin reported experiencing nausea. Elevated serum alkaline phosphatase levels were detected in four patients during the blood test [173].

Another case report was about a male patient, aged 74 years and afflicted with various medical conditions, who was admitted to the emergency department due to the presence of a sizable hematoma in his left thigh. The subject initiated a daily intake of 1 g of curcumin, in addition to the prescribed medications for his illnesses, one week prior. The researchers suggested that an elevated dosage of curcumin could potentially be linked to heightened antiaggregant and anticoagulant effects [174].

It has been found that curcumin is a potent chelator of iron [175]. According to in vivo research, adding curcumin to mice’s meals caused them to develop the symptoms of iron deficiency anaemia, including a drop in serum iron levels, a reduction in transferrin saturation, and the development of hypochromic red blood cells. Additionally, bone marrow and spleen iron levels may be decreased by curcumin [176].

According to the literature, a few other adverse effects of curcumin have been reported, indicating that the incidence of headache, skin rash, and yellow stool is dose-dependent and was observed in 7 of the 24 participants [170,177]. Additional adverse effects comprise gastrointestinal irritation, abdominal discomfort, hypersensitive dermatological responses [178], and triggered uterine contractions in pregnancy [179].

Table 2 offers an overview of all the health benefits of curcumin and the mechanisms behind them as described in recent literature sources.

## 6. Nanoscale Colloidal Systems for Curcumin Delivery

Microemulsions (MEs) are thermodynamically stable nanocolloidal systems usually consisting of at least three components: water, oil, and surfactant. These systems have the advantage of forming spontaneously because the surfactant lowers the free energy of the thermodynamic system [186]. Another advantage is that they are visually transparent, optically isotropic [187], and stable over a long period of time because their micelle sizes do not exceed 100 nm [188]. Winsor first classified the multiphase microemulsion systems as WI and WII; two-phase systems correspond to oil-in-water (O/W) microemulsions coexisting with an oil phase and water-in-oil (W/O) microemulsions coexisting with excess water, respectively. When the surfactant is concentrated in a middle phase that coexists with oil and water, the system is called WIII, and when the added surfactant leads to a single phase, it is called WIV [189].

Similar to microemulsions, nanoemulsions (NEs) are colloidal systems made up of water, oil, surfactants, and other adjuvants [190]. These systems present high surface area and kinetic stability against coalescence, making them suitable for various applications [191]. The classification of these systems is indicated by the distribution of the water and oil phases and by the synthesis procedure. Thus, there are simple nanoemulsions (one-step procedures) and multiple nanoemulsions (two-step procedures). Simple nanoemulsions can be divided into O/W nanoemulsions (dispersion of oil droplets in aqueous medium) and W/O nanoemulsions (dispersion of water droplets in oil medium). Multiple nanoemulsions can be categorized as water-in-oil-in-water (W/O/W) nanoemulsions and oil-in-water-in-oil nanoemulsions (O/W/O) [192].

The distinction between microemulsions and nanoemulsions is that microemulsions are thermodynamically stable, whereas nanoemulsions are kinetically stable [190]. Another difference is how these systems are obtained: microemulsions are formed spontaneously without introducing energy from the outer environment, while nanoemulsions need mechanical outer force to develop nanodroplets [193].

The formation of different types of systems depends on the proportion of components, the type of surfactant used, and the environmental conditions [194].

### 6.1. Preparation of Microemulsions and Nanoemulsions

Micro and nanoemulsions are produced by mixing two dispersed and continuous phases (oil and water) with an emulsifier (surfactant or surfactant/cosurfactant mix). The production of the desired system can be approached by low-energy methods in the case of both colloidal systems or by high-energy methods for nanoemulsions. Low-energy methods include phase titration and phase inversion, and high-energy methods include rotor-stator emulsification, ultrasonic homogenization, microfluidization, and high-pressure homogenization [193,195].

#### 6.1.1. Phase Titration Method

The water titration technique involves determining the ratios of oil/surfactant and, at times, cosurfactant and subsequently titrating them with a predetermined gradient of water. The determination of added water volume is recorded upon the detection of changes in phase number or physical characteristics, such as the presence of turbidity or gelation. After calculation of the relative quantities of the three constituents, a pseudo-ternary graph is constructed, wherein the boundaries demarcating each Winsor category are identified. The use of graphical representation facilitates the selection of the optimal water/oil/surfactant system for specific applications [196,197].

Ramalho et al. formulated a microemulsion through the process of water titration. The microemulsion was composed of isopropyl myristate as the oil phase, caprylocaproyl polyoxyl-8 glycerides as the surfactant, and water. The mean droplet size was 61.1 ± 10.3 nm with a polydispersity index (PDI) of 0.378 ± 0.1 and a zeta potential of −10.0 ± 2.1 mV [198]. Wang et al. prepared a microemulsion by water titration. The average particle size was 44.51 ± 0.83 nm, the pH was 5.57 ± 0.03, the electric conductivity was 32.4 ± 0.2 µS/cm, and the microemulsion was optically homogeneous and transparent [199]. Fernandez-Pena et al. developed stable microemulsion systems consisting of oleic acid as the oil phase, a mixture of alkyl polyglucoside and soybean lecithin as surfactants, and water using the water dilution method [200].

#### 6.1.2. Phase Inversion Method

The phase inversion technique involves altering the phase from O/W to W/O through the introduction of an excess amount of the dispersed phase [165] under constant temperature conditions (phase inversion composition, PIC) or by maintaining the composition constant while varying the temperature (phase inversion temperature, PIT) [201]. The introduction of a dispersed phase in PIC results in particle modification, thereby inducing a phase transition. Conversely, in PIT, a decrease in interfacial tension (upon cooling) triggers phase inversion [162].

Ee et al. utilized the temperature-phase inversion technique to fabricate a nanoemulsion. The resulting ultra-small droplet sizes ranged from 35 nm to 54 nm, with a low PDI of approximately 0.2 when stored at the optimal temperature [202]. Using the same method, Calligaris et al. created microemulsions that comprised diverse lipid phases and Tween 80 as a surfactant. Curcumin was incorporated into the lipid phase of a microemulsion, resulting in an observed enhancement of its chemical stability [203].

#### 6.1.3. Rotor-Stator Emulsification

The rotor-stator mixers are comprised of a stationary external stator and a mobile rotor [204,205]. As the rotor undergoes rotation, the emulsion is attracted towards the rotor head and subsequently expelled at a high velocity through the fixed rotor [206]. The reduction in particle size is caused by the high shear, stress, and grinding forces that arise from the interaction between the rotor and stator [206].

A stable oil-in-water nanoemulsion containing an aqueous phase rich in phenolic compounds was synthesized by Niknam et al. The rotor’s rotational velocity was measured at 20,000 rpm, while the duration of the mixing process was recorded as 10 min. The nanoemulsion exhibited a droplet size of 105.8 ± 10.3 nm and a PDI value of 0.255 ± 0.045 [207]. Using this technology, Rusanova et al. attempted to fabricate nanoemulsions and were successful in producing stable nanoemulsions [208]. Fuentes et al. also obtained nanoemulsions using this method, followed by an assessment of the impact of three different surfactants, namely whey protein isolate (WPI), soy lecithin, and Tween 20, on the resultant nanoemulsions. The authors of the study have determined that the physical stability of nanoemulsions is contingent upon the surfactant type, with the order of stability being soy lecithin < Tween 20 < WPI [209]. Scholz et al. achieved the production of a stable nanoemulsion for a minimum of three months through the use of a rotor-stator system. The stirring speed utilized in the production process was 36,000 rpm, and the duration of the production process was 5 min. The authors noted that implementing a rotor with an ultrafine slit size has the potential to decrease processing time [210].

#### 6.1.4. Ultrasonic Homogenization

This technique involves the production of small droplets through their exposure to high-intensity acoustic waves [211]. Section 3.3 already provided the essential information regarding the action of ultrasounds and corresponding phenomena. The aforementioned approach confers benefits due to its low surfactant consumption [212,213], non-toxicity, safety, and eco-friendliness [214,215]. Additionally, the resultant particles exhibit small size and long-term stability in terms of polydispersity index [214,216].

In one study, Guzman et al. utilized this method to produce nanoemulsions, wherein the oil phase consisted of *P. edilus* var. edilus seed oil (PEO) and a blend of sorbitan trioleate and polysorbate 80 was employed as the surfactant. The optimal conditions for emulsification were determined to be an ultrasonic power of 85.28 W and an irradiation time of 5.96 min. The study reported that the mean droplet size was 130 nm, the zeta potential was −30.4 mV, the PDI was 0.202 ± 0.015, the viscosity was 3.04 ± 0.17 cP, the pH was 6.82 ± 0.42, and the conductivity was 0.74 ± 0.18 mS/cm [217]. Eucalyptus oil nanoemulsions were prepared by Song et al. [216]. Their study determined that the most favorable process parameters were a sonication distance of 0.9 cm, a sonication amplitude of 18%, and a sonication time of 2 min. The measured particle size was found to be 18.96 ± 4.66 nm, with a polydispersity index of 0.39 ± 0.09 and a zeta potential of −31.17 ± 2.15 mV. The utilization of ultrasonic preparation techniques for eucalyptus oil nanoemulsion resulted in an enhancement of its activity against *E. coli* [216]. Alam et al. utilized the ultrasonic homogenization method to synthesize a nanoemulsion (CEO-NE) containing essential oil derived from *Cinnamomum cassia* (CEO). The synthesized CEO-NE exhibits a uniform morphology, characterized by a polydispersity index (PDI) of 0.380 and a mean particle diameter of 221.8 nanometers. The nanoemulsions that were obtained exhibited greater efficacy against *Klebsiella pneumoniae* in comparison to CEO. In addition, the efficacy of CEO-NE was evaluated against A549, an alveolar lung adenocarcinoma cell line, resulting in IC50 values of 50.12 and 18.05 for CEO and CEO-NE, respectively [218].

#### 6.1.5. Microfluidization

Microfluidization of the emulsions is performed using a microfluidizer [219]. The operational mechanism of the aforementioned system is contingent upon the intricate dynamics of microchannels that have been purposefully engineered for this purpose [220]. The fluid undergoing homogenization is pushed through the interaction chamber, consisting of microchannels, with the aid of a positive-dispersion pump. The fluid traverses tight channels and strikes a substrate, causing significant kinetic energy to perturb the structures, leading to the production of reduced-size particles [219]. The final particle size is influenced by various factors, such as the number of passes, treatment pressure, and material properties [221].

Tocotrienol-enriched nanoemulsions were developed by Goh et al. utilizing various surfactants. The results of the experiment indicate that following 10 homogenization cycles with incremental pressure, a reduction in droplet size from 120 nm to 65.1 nm was observed. Nanoemulsions stabilized only with Tween series surfactants or an emulsified blend of Brij 35 and Span 80, homogenized at 25,000 psi and 10 cycles, yielded droplet sizes below 100 nm and a narrow size distribution, with a PDI value less than 0.2. However, the combination of Tweens with Span 80 resulted in the formation of nanoemulsions characterized by droplet sizes exceeding 200 nm [222]. Kim et al. conducted a study where they obtained stable nanoemulsions containing vitamin A and vitamin C. The particle sizes of the nanoemulsions were approximately 475.7 and 225.4 nm, while the zeta potentials were approximately −33.5 and −21.3 mV [223].

#### 6.1.6. High Pressure Homogenization

The process of homogenization under high pressure occurs inside a homogenizer, which is best suited for fluids possessing low to intermediate viscosities [224]. Prior to homogenization, a preliminary mixture comprising oil, water, surfactant, and/or cosurfactant is generated [224,225]. The initial rough mixture is inserted into the homogenizer and subsequently propelled through various nozzles of varying diameters with the aid of a piston. The fragmentation of large droplets into smaller ones is caused by shear forces, turbulence, and cavitation [224]. The final product is dependent on various factors, including the diameter of the nozzle, the viscosity of the emulsion, the number of passes, and the homogenizing pressure [226]. The method is deemed advantageous owing to the low consumption of surfactant, short emulsification time, and stability of the resulting nanoemulsion [227].

A nanoemulsion containing Sichuan pepper essential oil was developed by Shi et al. The results indicated that the system exhibited favorable stability over the course of the study. Specifically, the average particle size and zeta potential of the nanoemulsion underwent very small changes over time, from 125.07 nm and −33.12 mV to 134.53 nm and −29.27 mV, respectively [227]. In their study, Hidajat et al. [226] successfully produced a small-sized limonene nanoemulsion under a pressure of 1000 bar following five passes. The average particle size was 55 nm. The nanoemulsion exhibited remarkable stability for a period of 28 days [226]. Yakoubi et al. conducted a study wherein they developed a new nanoemulsion through the high-pressure homogenization method. The optimal nanoemulsion exhibited a droplet size of 270 nm, an interfacial tension of approximately 11 mN/m, and a zeta potential of −15 mV [228]. The results indicated that the nanoemulsion was stable under various conditions. The physical and chemical stability of nanoemulsions loaded with D-limonene was investigated by Sohan et al. The physical stability of the nanoemulsion was observed over a period of 30 days, wherein the droplet size range remained consistent within the range of 122–130 nm [229].

**Figure 4:**
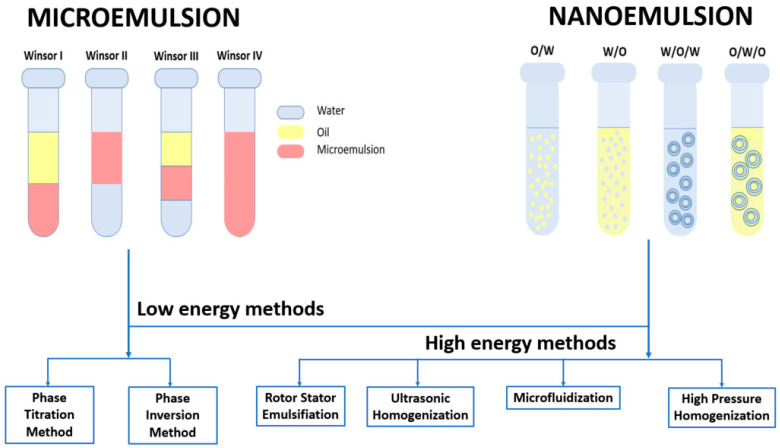
Types and methods of synthesis for microemulsions and nanoemulsions.

Figure 4 displays the existing types of microemulsions and nanoemulsions and the low- and high-energy methods employed for their fabrication.

### 6.2. Microemulsions and Nanoemulsions for Curcumin Delivery

As described in Section 5, curcumin exhibits numerous advantageous effects on human health; however, because of its poor bioavailability, its broad variety of potential uses has not been fully explored. The primary contributors to low bioavailability include the poor solubility of this bioactive compound, its limited absorption in the gastrointestinal tract as a result of its hydrophobic nature, its quick clearance from the systemic circulatory system, and its restricted ability to cross the blood-brain barrier [230]. Once curcumin is taken orally, it goes through a significant number of metabolic steps, which result in sulfate and glucuronide conjugates becoming its major molecular forms in the body [231]. Both the autooxidation event that takes place at physiological pH and the degradation reaction make significant changes to the structure and pharmacological behavior of curcumin, which severely restricts its use in medical applications [106].

In order to improve the water solubility [232] and dispersibility and to lengthen the self-life of the bioactive substance, such a lipophilic compound as curcumin can be encapsulated into colloidal carriers with tiny droplet sizes, as are microemulsions and nanoemulsions [233]. Due to their increased O/W surface, they encourage interactions of the encapsulated compound with biological membranes and intestinal enzymatic reactions, resulting in improved bioavailability and improved dissolution and solubility rates. They also effectively undergo mass transfer through the mucus of the intestinal epithelium [234] and permit a sustained or controlled release for parenteral, transdermal, oral, nasal, intravenously, ophthalmic, and other delivery paths [235].

Considering the several therapeutic properties of curcumin, numerous efforts have been made to obtain stable colloidal systems and observe their beneficial actions with respect to compound delivery to targeted organs. These recent studies are summarized in Table 3, which shows the types of MEs and NEs that have been prepared to deliver curcumin, the components used for fabrication, the average particle size attained, and the effects elicited by the encapsulation in terms of curcumin bioavailability and bioactivity.

## 7. Conclusions

Turmeric is a perennial plant valued not just for the flavor and color it adds to meals but also for the curcumin it contains and its numerous health benefits.

Among these, curcumin is known as an effective antioxidant because it can reduce mitochondrial oxidative stress by increasing the effects of superoxide dismutase, glutathione, and catalase. It also alleviates depression because it inhibits monoamine oxidase enzymes, modulates various neurotransmitters, and acts as an anti-inflammatory agent. Diabetes can be efficiently treated with curcumin by increasing insulin resistance and decreasing leptin, resistin, and insulin levels. Recent research has also indicated that curcumin exhibits potent antibacterial properties and holds potential as an anticancer agent.

The limited efficacy of curcumin can be attributed to its rapid metabolism and degradation in the presence of physiological factors. Primarily due to its high hydrophobicity, this bioactive compound cannot reach its full therapeutic potential through ingestion or other routes of administration; however, its encapsulation in colloidal micro/nanoemulsion systems presents a viable alternative for protecting the bioactive substance and facilitating optimal absorption. Owing to their small dimensions, such systems afford versatility in administration routes, enable regulated release, and promote the compound’s interaction with biological membranes, thereby enhancing its bioavailability.

## Figures and Tables

**Table 1:** Extraction methods for curcuminoids from plant material and their corresponding yields reported in the recent literature.

Extraction Method	Extraction Time	Solvent Used	Additional Technical Information	Extraction Yield	Reference
Soxhlet Extraction	48 h	Ethanol	40 °C	1.25%	[74]
UAE	2 h	Ethanol	40 °C, 1:30 (*w*/*v*), 240 W, 22 kHz	73.18%	[53]
UAE	1 h	Ethanol	35 °C, 250 W, 22 kHz	72%	[75]
MAE	29.99 min	Ethanol	160 W, 1:20 (*w*/*v*)	10.32%	[62]
MAE	9 min	Ethanol	900 W	88%	[76]
EAE	8 h	Water	Enzyme: α-amylase, incubation time: 5 h	26.04%	[77]
PLE	20 min	Ethanol	60 °C, 100 bar	99 ± 5%	[73]
EAE	8 h	Water	Enzyme: cellulase, 40 °C, pH: 4.5–6	25–27%	[78]
MAE	2 min	Acetone	100 W	82.4%	[79]
UAE	40 min	Ethanol	1:10 (*w*/*v*), 240 W, 42 kHz	7.67 ± 1.36%	[59]
Soxlet extraction	7 h	Acetone	56.53 °C	4.09%	[80]
EAE	1 h	Water	Enzyme: pectinase, pH: 5, 50 °C	8.36%	[19]
MAE	2 min	Acetone	300 W	3.72%	[55]
Soxhlet Extraction	8 h	Acetone	60 °C	6.9%	[55]
PLE	5 min	Water/Ethanol 50/50	130 °C, 1:20 (*w*/*v*)	15.8%	[72]
SFE	1 h	SC-CO_2_	70 °C, 425 bar	0.68–0.73%	[69]
UAE	40 min	Ethanol	240 W; 42 kHz, 1:30 (*w*/*v*)	16.03%	[59]
SFE	2.5 h	SC-CO_2_	47 °C, 260 bar	71%	[81]

**Table 2:** Health benefits of curcumin and the mechanisms by which it affects them according to recent literature.

Effect of Curcumin	Mechanism of Action	Reference
Antioxidant	Can scavenge reactive oxidizing substances	[180]
	Enhances the activities of superoxide dismutase (SOD), catalase (CAT), and glutathione peroxidase (GPx)	[116]
	Activates antioxidant factors including Nrf2, heme oxygenase (HO-1), and proliferator-activated receptor-gamma coactivator (PGC-1α)	[181]
	Prevents LDL (low-density lipoprotein) oxidation	[182]
Antibacterial	Penetrates bacterial cell membranes and makes them permeable for antibiotic absorption.	[119]
	Causes phototoxicity and inhibits bacterial growth when exposed to blue light.	[15]
	Inhibits the growth of Gram-negative and Gram-positive bacteria	[121]
Antidepressant	Can regulate the concentrations of diverse neurotransmitters, including norepinephrine, dopamine, and serotonin.	[136]
	Has strong interactions with the MAO enzyme	[183]
Antidiabetic	Can increase Akt and GSK-3β phosphorylation	[141]
	Can lower glucose levels and decrease insulin resistance, dyslipidemia, and malondialdehyde levels	[184]
	Can enhance the expression of the *GLUT4* gene	
	Can reduce the levels of leptin, resistin, interleukin (IL)-6 IL-1β, and tumour necrosis factor-α	[145]
Anticancer	Inhibits the proliferation of human colon cancer SW620 and MC-38 cells	[165]
	Has antiproliferation effects by inhibiting the binding activity of NF-kB, AP-1, EGR, and β catenin	[185]
	Inhibits liver cancer HepG2 cells by inhibiting heat shock protein 70 (HSP70)—toll-like receptor 4 (TLR4) signaling	[149]
	Can inhibit the migration of esophageal cancer tumor cells by enhancing the expression of nonmetastatic gene 23 (*Nm 23*), antimetastatic protein tissue inhibition metalloproteinase (TIMP)-2, and E-cadherin	[160]

**Table 3:** Microemulsions and nanoemulsions for curcumin delivery reported in the recent literature.

Type of System	Particle Size (nm)	Components	Effect	Reference
O/W ME	10.07 ± 0.45	Vitamin E, Tween 20, Ethanol, Water	Improve solubility and stability and oral uptake of curcumin	[236]
O/W NE	23.23 ± 2.86	IPM, Tween 80: Glycerol, Surfactin	Improve bioavailability, inhibit activity of Caco2 cell	[237]
O/W ME	21.81 ± 0.20	Miglyol 812 N, Water, PEG 30 castor oil:Span 80	Improve solubility, stability and modified release of curcumin at gastrointestinal tract pHs	[238]
O/W ME	80.02 ± 3.29	Soybean Oil, Lecithin:Tween 80, Water	Enhance anti-Hep G2 activities	[239]
O/W ME	71.8 ± 2.45	Ethyl oleate, Lecithin/Tween 80, Water	Prevent degradation process of curcumin, enhance the skin permeation	[240]
O/W ME	27.3 ± 2	Capryol 90, Cremophor RH 40, Transcutol P	Enhance the oral bioavailability	[241]
O/W NE	174.44	HI-Cap 100, Sunflower oil, Water	Enhance solubility of curcumin	[242]
O/W ME	8	Ethyl butyrate, Pluronic surfactant F127, Fetal bovine serum, Sodium Caprylate	Enhance hepatoprotective, neuroprotective and anti-cancer effects of curcumin	[243]
O/W NE	100–900	Cottonseed oil, Ethanol, Glycerol, Poloxamer, Tween 20, SDS DTAB	Enhance antioxidant efficacy of curcumin	[244]
O/W NE	372	Pluronic 127, Olive Oil, Water	Inhibit MDA-MB-231 breast cancer cell	[245]
O/W NE	141.6 ± 15.4	WPC-70, Tween 80, MCT-60, Water	Increase hydrophilicity and bioaccessibility of curcumin	[246]
O/W NE	218	Span 20 saturated MCT, Monostearin, water, Tween 20	Improve oral bioavailability of curcumin	[247]
O/W NE	26.76 ± 0.9	PEG 400/Tween 80/Water	Accelerate the skin tissue regeneration process	[248]
O/W NE	105	Soybean oil, MCT, Lecithin, Benzyl alcohol, Sodium Oleate	Improve plasma resistance of curcumin	[249]
O/W NE	102–131	PEG-660 stearate, castor oil, purified fish oil, egg lecithin, water	Allow intranasal administration of curcumin	[250]
O/W NE	130	Tween 80, Ethanol, Propylen Glycol, Myrrh Oil	Improve anti-inflammatory effects of curcumin	[251]
O/W ME	10.9	Soybean Oil, Tween 80, Ethanol, Water	Enhance curcumin potential to inhibit growth of colon cancer cells HT-29	[252]
O/W NE	85 ± 1.5	Glyceryl monooleate, Cremophor PH40, Polyethylene glycol 400, Water	Improve curcumin permeability through skin and protect curcumin from chemical degradation	[253]
O/W NE	34.52 ± 2	MCT, Cremophor RH 40, Glycerol, Water	Enhance cellular cytotoxicity, cellular uptake, cell cycle arrest and apoptosis against prostate cancer cells	[254]
O/W ME	66.74 ± 3.46	Capmul MCM, Accenon CC: Transcutol P, Aqueous polycarbaphil	Improve transnasal delivery of curcumin	[255]

## Data Availability

No new experimental data were created in this study. Data sharing is not applicable to this review article.
